# Supplementary material for: Navigating mental health needs at sea: Perspectives from the Canadian maritime community during the COVID-19 pandemic
Source: PLOS Glob Public Health. 2026 May 19;6(5):e0006493. doi: 10.1371/journal.pgph.0006493 (PMC13186379; doi:10.1371/journal.pgph.0006493)
Supplement: Attachment — Submitted filename: Response to Reviewers.docx [file pgph.0006493.s002.docx]

**Dear reviewers,**

Our most sincere thank you for your observations, which have strengthened the manuscript. Detailed responses to each comment are available in the table below.

Warmly,

The research team

| **Comments** | **Response** |
| --- | --- |
| The mental health of seafarers is an important issue that does not receive enough attention. Research in this area is therefore very welcome. However, this manuscript has several important shortcomings, many of which have already been clearly identified by the reviewer. In particular, the discussion section would benefit from deeper engagement with the findings and a clearer connection to existing international research on maritime mental health. | Thank you for this suggestion. In response, we strengthened the Discussion to more explicitly situate our findings within international maritime mental health research and to engage more directly with our data. |
| In the ethics statement in the Methods, you have specified that verbal consent was obtained. Please provide additional details regarding how this consent was documented and witnessed, and state whether this was approved by the IRB. | Thank you for raising this point, the procedure to document consent and it’s IRB approval have now been further specified. |
| Please provide a detailed online Financial Disclosure statement. This is published with the article. It must therefore be completed in full sentences and contain the exact wording you wish to be published.  a) State the initials, alongside each funding source, of each author to receive each grant. For example: “This work was supported by the National Institutes of Health (####### to AM; ###### to CJ) and the National Science Foundation (###### to AM).”  b) State what role the funders took in the study. If the funders had no role in your study, please state: “The funders had no role in study design, data collection and analysis, decision to publish, or preparation of the manuscript.”  For more information, please go to our submission guidelines: https://journals.plos.org/globalpublichealth/s/submission-guidelines#loc-financial-disclosure-statement | Thank you, implemented. |
| Please ensure that the funders and grant numbers match between the Financial Disclosure field and the Funding Information tab in your submission form. Note that the funders must be provided in the same order in both places as well. | -- |
| Please update your online Competing Interests statement. If you have no competing interests to declare, please state: “The authors have declared that no competing interests exist.” | -- |
| 5. In the online submission form, you indicated that "The data that support the findings of this study are available from the corresponding author upon reasonable request.".  All PLOS journals now require all data underlying the findings described in their manuscript to be freely available to other researchers, either  a) In a public repository,  b) Within the manuscript itself, or  d) Uploaded as supplementary information.  This policy applies to all data except where public deposition would breach compliance with the protocol approved by your research ethics board. If your data cannot be made publicly available for ethical or legal reasons (e.g., public availability would compromise patient privacy), please explain your reasons by return email and your exemption request will be escalated to the editor for approval. Your exemption request will be handled independently and will not hold up the peer review process, but will need to be resolved should your manuscript be accepted for publication. One of the Editorial team will then be in touch if there are any issues.  For further assistance, you may go to: http://journals.plos.org/globalpublichealth/s/data-availability | Due to the nature of the qualitative interviews, including a small number of key informants in identifiable roles, public sharing of full interview transcripts would create a risk of deductive disclosure (re-identification) and would breach compliance with the protocol approved by the Interdisciplinary Committee on Ethics in Human Research (ICEHR) at Memorial University of Newfoundland. Therefore, the interview transcripts are not publicly available. Additional access to de-identified qualitative data will be granted upon request. |
| 6. If the reviewer comments include a recommendation to cite specific previously published works, please review and evaluate these publications to determine whether they are relevant and should be cited. There is no requirement to cite these works unless the editor has indicated otherwise. | -- |
| 1. Insufficient reflexivity and researcher role specification: The study provides limited information on the researchers’ positionality, disciplinary background, or prior engagement with maritime or mental health sectors. lack of clarity on researcher's positionality opens the door to researchers’ assumptions shaping the data collection or interpretation, lack of information n power dynamics in interviews (e.g., seafarers vs key informants), or how insider/outsider status influenced rapport and disclosure. In qualitative research, particularly with marginalized workers, reflexivity centered on researcher positionality is central to trustworthiness. Its absence weakens claims of analytical rigor. Implication: Readers are left uncertain about how meaning was co-constructed and how interpretive decisions were made. Address: this paper: Goundar, P. R. (2025). Researcher Positionality: Ways to Include it in a Qualitative Research Design. International Journal of Qualitative Methods, 24. https://doi-org.proxy.bib.uottawa.ca/10.1177/16094069251321251 is a good guide on how and where to include researcher positionality. This reviewer feels that including researcher positionality, how it has shaped the data collection and interpretation of the findings and the final conclusion is a necessary requirement in qualitative research and encourages the researchers to include in their paper. This point reflects need for a major revision. This reviewer recommends to the researchers to look at the COREQ domain 1 (research team and reflectivity) points and include all of them in their paper. | We thank the reviewer for highlighting the importance of researcher positionality and reflexivity. In response, we have added a new subsection titled *“Researcher Positionality and Reflexivity”* within the Methods section. This section outlines the first author’s disciplinary background, positioning, and prior engagement with the topic. It also clarifies how reflexive practices—including field notes, peer debriefing and review of interview guides—were used to address power dynamics, mitigate assumptions, and enhance analytical rigor. |
| 2. Points for minor revisions: a) participant recruitment pathways are insufficiently detailed (e.g., via employers, unions, welfare orgs).b) No discussion of refusals, dropouts, or reasons for non-participation. Important given potential fear of reprisal among seafarers. c) context of data collection (setting-where the participants on leave or on board for example) during the interview is critical for interpreting disclosure/power dynamics. | Thank you for these comments, they have been implemented by adding more details on recruitment, refusals, and dropouts, as well as the setting of data collection. |
| 3. Limited engagement with global mental health research: This reviewer will encourage researchers to include a discussion point on how their current research engages with larger global mental research ( There is a mention on how it aligns globally (lines 588-590) ), but the current discussion is largely positioned on national policy and frameworks. Globally, organizations such as The mission to Seafarers, International Seafarers’ Wellness and Assistance Network, and the International Transportation Workers’ Federation, among others, have created wellness directories, multilingual helplines etc. The larger global mental health community will benefit from researchers commenting on how and what this body of research adds to it. Current implications section is focused on National level impact on policy and practice. | Thank you for highlighting the need to more explicitly situate this study within global mental health and the international seafarer welfare ecosystem. The Discussion and Implications sections have been expanded to (i) acknowledge established global maritime supports and (ii) clarify what this study adds to global mental health scholarship. The revised text also explains how Canadian service-delivery assets discussed in the paper (MHFA; Stepped Care 2.0) can be translated into exportable implementation strategies. |
